# Job satisfaction of hospital pharmacists in a representative province in Mekong Delta, Vietnam

**DOI:** 10.1371/journal.pone.0291201

**Published:** 2023-09-14

**Authors:** Van De Tran, Thi My Loan Vo, Khanh Nguyen Di, Quang Loc Duyen Vo, Rebecca Susan Dewey, Trung Tin Pham, Ba Kien Tran, Duy Toan Pham

**Affiliations:** 1 Department of Health Organization and Management, Can Tho University of Medicine and Pharmacy, Can Tho, Vietnam; 2 Faculty of Pharmacy, Can Tho University of Medicine and Pharmacy, Can Tho, Vietnam; 3 Tam Binh District Health Center, Vinh Long, Vietnam; 4 Department of Medical Testing, Faculty of Health Sciences, Dong Nai Technology University, Dong Nai, Vietnam; 5 Department of Chemistry, College of Natural Sciences, Can Tho University, Can Tho, Vietnam; 6 Sir Peter Mansfield Imaging Centre, School of Physics and Astronomy, University of Nottingham, Nottingham, United Kingdom; 7 Department of Pharmaceutical Administration, Hai Duong Central College of Pharmacy, Hai Duong, Vietnam; Nova Southeastern University / Yarmouk University, UNITED STATES

## Abstract

**Background:**

Job satisfaction is an important factor affecting job performance and turnover of healthcare workers, especially hospital pharmacists. Nevertheless, limited studies have reported this issue in the context of Vietnam.

**Objectives:**

To help maintain the quality and size of the limited hospital pharmacy workforce in Vietnam, especially in the Mekong Delta area, this study investigated the job satisfaction of hospital pharmacists, and the associated factors, in Vinh Long province, a representative province in the central Mekong Delta.

**Methods:**

A cross-sectional survey was conducted, recruiting hospital pharmacists working in all 17 province/district-affiliated healthcare facilities across Vinh Long province, Vietnam, between August and September 2022.

**Results:**

Among the 235 survey participants (representing a response rate of 97.1%), 189 pharmacists (80.4%) reported that they were satisfied with their job. Working conditions, leadership styles, and benefits were factors found to significantly influence job satisfaction. Pharmacists who had worked in the field for 3–5 years (OR = 3.752, 95% CI = 1.036–13.595), more than 5 years (OR = 6.361, 95% CI = 2.264–17.875), did not have additional duties besides their primary responsibilities (OR = 2.046, 95% CI = 1.005–4.163), and worked in a private healthcare facility (OR = 12.021, 95% CI = 1.470–98.316), were significantly more likely to be satisfied with their job.

**Conclusions:**

Most hospital pharmacists were satisfied with their current job. To further improve job satisfaction in this population, further improvements to working conditions are necessary.

## Introduction

Hospital pharmacy is a healthcare service provided by hospital pharmacists, comprising the preparation, storage, distribution, and dispensation of drugs, medicines, and medical devices, in addition to giving instructions for their uses to patients, doctors, and other healthcare professionals, and conducting other administration and documentation tasks [1]. The hospital faculty/department of pharmacy is a specialized department, typically under the direct management of the hospital director, who is responsible for the management of all hospital pharmacists and pharmacy-related workload [2, 3]. The work of a hospital-pharmacist includes, but not limited to, planning for drug supply, providing drug information, administering and monitoring the import/dispensing/storage of drugs, implementing the activities of the drug and treatment council, performing clinical pharmacy consultations, monitoring drug usage, participating in pharmacovigilance, reporting information related to adverse drug effects, and conducting scientific research and training [2, 4]. Due to the diversity of this workload, a hospital pharmacist has no easy time performing these tasks rapidly and efficiently, resulting in workload pressure, stress, depression, and reduced job satisfaction [3, 5–8]. According to the International Federation of Pharmaceuticals (FIP), there has been a significant decrease in job satisfaction in hospital pharmacists and pharmaceutical-industry workers worldwide [9, 10]. Job dissatisfaction is associated with further negative consequences, such as increased absenteeism, stress, fatigue, and reduced effort, which can lead to decreased work quality and increased staff turnover [10–12]. To maintain the size and quality of the healthcare workforce in low-income countries such as Vietnam, it is crucial to investigate job satisfaction among hospital pharmacists and identify factors associated with high and low job satisfaction.

Vinh Long province is located in the central Mekong Delta in the South of Vietnam, with a total area of 1,525 km^2^ and a population of approximately 1 million people [13, 14]. There are approximately 3,378 medical staff including 384 pharmacy staff, and provided approximately 3.5 million medical examinations and treatment appointments in 2019 in Vinh Long province [15]. The organizational structure of the Vinh Long healthcare system is inconsistent, with inadequate human resources, both in quantity and quality [15], and a large number of healthcare professionals leaving their jobs and/or moving to the private sector [16]. Between January 1st, 2021, and June 30th, 2022, a total of 9,680 healthcare workers resigned in Vietnam [16]. Furthermore, the problem was getting worse, as the number of healthcare workers who resigned from positions in Vinh Long in the first six-months of 2022 was nearly equal to that in the whole of 2021 [17]. As mentioned previously, one of the main reasons for resigning is low job satisfaction [18]. Nevertheless, although the job satisfaction of hospital pharmacists has been investigated in Australia [19], Ethiopia [7], South Africa [20], Saudi Arabia [21], and China [22], no such studies have been conducted in the Mekong Delta, Vietnam. Therefore, this study aimed to assess the level of satisfaction and analyze the factors associated with job satisfaction of hospital pharmacists in Vinh Long as a representative province of the Mekong Delta, Vietnam.

## Methods

### Study design

**Fig 1:**
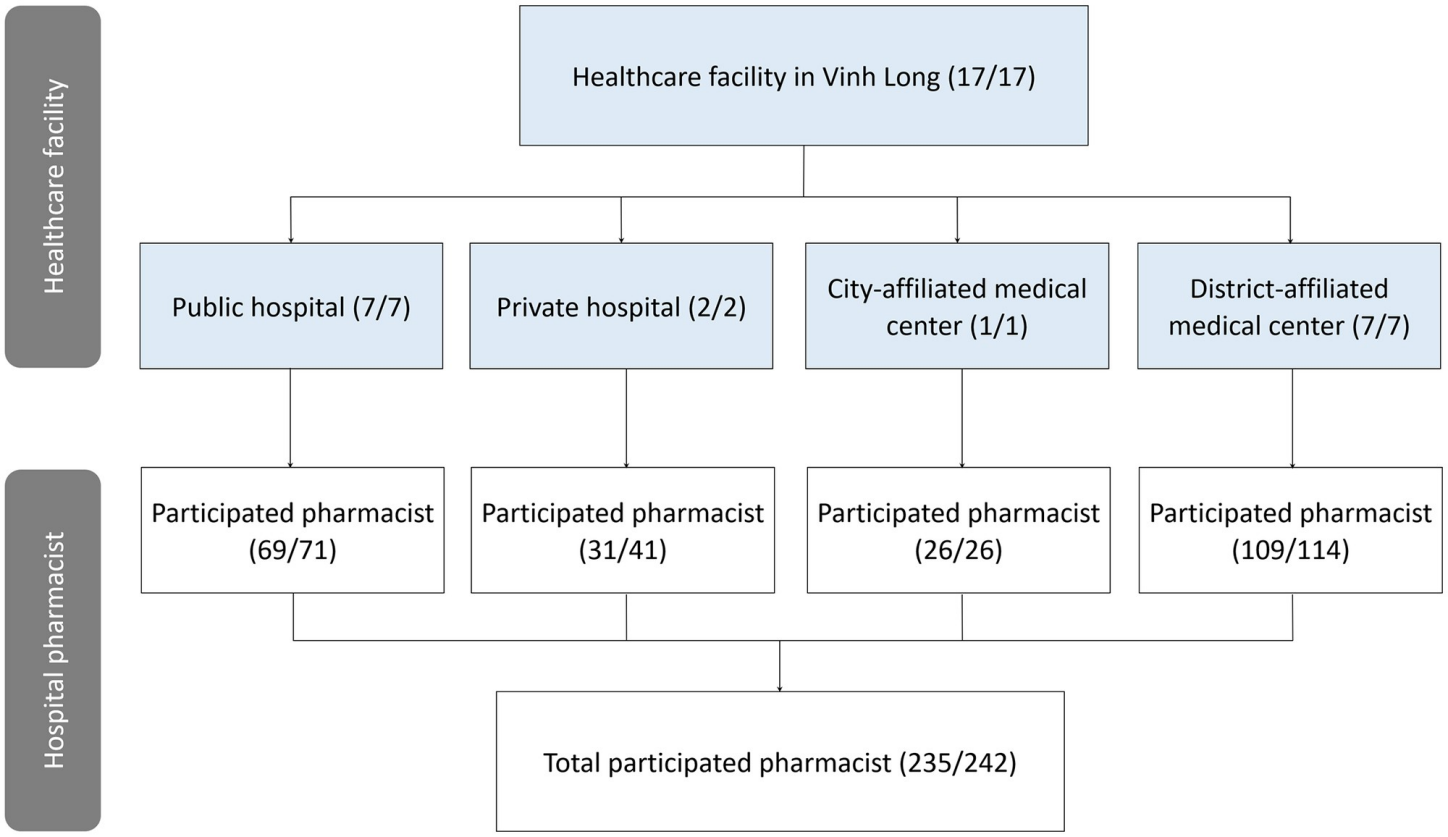
Flowchart of participant recruitment. Numbers in parentheses (x/y) indicate the participated healthcare facilities/hospital pharmacists (x) over the total facilities/pharmacists in the Vinh Long province (y).

The study was conducted using a cross-sectional survey administered between August 1st and September 30th, 2022, in Vinh Long province, Vietnam. Vinh Long province was purposely chosen as the research sites among 63 provinces/cities in Vietnam to represent the Mekong Delta region. As of 2022, Vinh Long has a total of 115 commune health stations, which are primary healthcare centers, and 17 province/district-affiliated healthcare facilities. However, only province/district-affiliated healthcare facilities were selected in this study (Fig 1), including 7 province-affiliated public hospitals (Civil military regional general hospital, Hoa Phu general hospital, Vinh Long general hospital, Vinh Long eye hospital, Vinh Long lung hospital, Vinh Long psychiatric hospital, and Vinh Long traditional medicine hospital), 2 province-affiliated private hospitals (Trieu An—Loan Tram hospital and Xuyen A Vinh Long general hospital), 1 city-affiliated medical center, and 7 district-affiliated medical centers (Binh Tan, Long Ho, Mang Thit, Vung Liem, Tam Binh, Tra On, and Binh Minh). All hospital pharmacists working in these facilities were recruited to participate in the current study. Pharmacists who were on maternity leave or attending long-term training were excluded from the study. The term ’hospital pharmacists’ in our study refers to healthcare professionals who have completed a pharmacy degree at intermediate, college, university, and postgraduate levels, and work in the pharmacy department and/or medicine outlets within province/district-affiliated healthcare facilities settings. This study excluded pharmacy professionals from hospital pharmacies/medicine outlets that were leased to external entities for business purposes because these pharmacy professionals operated independently of the healthcare facility’s management, despite the physical location of the pharmacy/medicine outlet within the healthcare facility premises. In the context of Vinh Long specifically, and Vietnam in general, both public and non-governmental healthcare facilities require pharmacists to have a minimum educational qualification of a college/intermediate degree or higher. They are subjected to supervision and management to ensure safety and quality standards, and they receive support and communication with their colleagues. However, the salary and benefits may vary, with non-governmental healthcare facilities often offering higher salary levels [23]. Workload can also differ, with public healthcare facilities often facing greater work pressures [24].

### Questionnaire

A self-administered questionnaire comprising two parts was distributed in printed format. The first part included four dimensions: (1) the work environment, (2) leadership and colleagues, (3) internal regulation, income, and benefits, and (4) work, training, and promotion opportunities. Each dimension comprised several items (37 items across the four dimensions), representing issues that may affect hospital pharmacist job satisfaction. Seven additional items for assessing the level/degree of overall job satisfaction (OJS) were also included (OJS 1: Feeling proud to work at the hospital, OJS 2: Achieving personal successes while working at the hospital, OJS 3: Trusting in the future development of the hospital, OJS 4: Willing to stay long-term in the current department, OJS 5: Willing to stay long-term at the hospital, OJS 6: Overall satisfaction with the hospital leadership, and OJS 7: Self-assessment of job performance at the hospital). The items included in this section were designed based on the job satisfaction assessment tool published by the Vietnam Ministry of Health, issued together with Decision 3869/QD-BYT [25]. Participants were asked to rate each item or statement using a 5-point Likert scale ranging from 1 (strongly disagree) to 5 (strongly agree).

The second part of the questionnaire captured the sociodemographic characteristics of the respondent, including gender (man and woman), age (< 30 years, 30–40 years, and > 40 years), accommodation type (rental house and private house), marital status (single and married), family members (≤ 3 and > 3), and average monthly income (< 5 million Vietnamese dong [VND] and ≥ 5 million VND), in addition to job-related information, such as working years (< 3 years, 3–5 years, and > 5 years), highest academic degree (college/intermediate, university/bachelor, and master/specialist level I), presence of additional duties besides primary responsibilities (no and yes), monthly on-duty times (< 4 and ≥ 4), overtime work (no and yes), and hospital type (public and private). In addition, participants were also asked about their workload, which encompassed a wide range of activities like administration, pharmacy official work, drug storage and distribution, pharmacy statistics, clinical pharmacy, health program implementation, outpatient drug distribution, and others.

To ensure comprehensive data collection, printed questionnaires were directly distributed to all hospital pharmacists based on a comprehensive list obtained from province/district-affiliated healthcare facilities. Prior to this process, hospital leadership was approached via phone to obtain their consent for survey implementation, and all hospital leaders agreed to participate. Subsequently, the research team personally delivered the printed questionnaires to the hospital pharmacists after departmental meetings, and the completed questionnaires were collected afterwards. For those who were unavailable during the survey period, the questionnaires were sent back to their colleagues for delivery and collected by the end of that week. A pilot survey was conducted in one hospital (Tam Binh medical center) to assess the clarity and accuracy of the questionnaire content. Subsequent modifications were not deemed necessary as participants stated that the questions were clear and unambiguous. The survey took between 5 and 7 minutes to complete.

### Data analysis

Data were processed using IBM SPSS version 25 (IBM, Chicago, IL, USA). Frequencies and percentages were used to present categorical variables, while mean (standard deviation, SD) and median (interquartile range, IQR) were used to present continuous variables.

Principal component analysis using the Promax method with Kaiser normalization was used to determine the latent factors of the structure consisting of 37 items with varying degrees of association with job satisfaction. Five extracted components with eigenvalues greater than one were retained. Acceptable items were defined as those possessing a factor loading of more than 0.5 on the primary factor and no cross-loadings (the next highest loading various by at least 0.2) [26, 27]. The first factor rotation round removed 6 items (JS 35, JS 33, JS 20, JS 37, JS 32, and JS 27, where "JS" is an abbreviation for job satisfaction). The second round removed 4 additional items (JS 34, JS 36, JS 21, and JS 31). The third round removed item JS 19. The remaining items are shown in Table 1. The fourth round yielded 5 factors: (1) working conditions, (2) leadership styles, (3) income, (4) relationship with colleagues, and (5) benefits (Table 2). The Cronbach’s alpha of the 5 resulting factors and the overall satisfaction scale were greater than 0.7, indicating a high degree of reliability (Table 2).

**Table 1:** Standardized loadings of items for each factor. Factor 1: working conditions; factor 2: leadership styles; factor 3: income; factor 4: relationships with colleagues; factor 5: benefits.

Item no.	Items/Statements	Factor 1	Factor 2	Factor 3	Factor 4	Factor 5
JS 1	The workplace is spacious, clean and airy	0.88				
JS 7	The working environment is safe for the hospital pharmacists	0.82				
JS 2	Outdated office equipment such as desks and chairs are replaced in a timely manner	0.76				
JS 9	Patients and their family members respect and cooperate with hospital pharmacists during treatment	0.75				
JS 8	The hospital ensures security and order for hospital pharmacists	0.75				
JS 5	Personal protective equipment for hospital pharmacists is complete and ready to use	0.72				
JS 3	There are rooms-on-duty for hospital pharmacists	0.71				
JS 6	The learning environment facilitates for hospital pharmacists (i.e., library, reading room, internet access) are adequate	0.61				
JS 4	There is a reasonable assignment of time-on-duty and overtime work	0.59				
JS 13	Leaders are open-minded		0.87			
JS 14	Leaders praise and motivate hospital pharmacists for on-time task completion and work progression		0.85			
JS 12	Leaders care, respect, and treat hospital pharmacists equally		0.85			
JS 11	Leaders assign work in accordance with employees’ expertise		0.83			
JS 10	Leaders have the ability to handle, administer, and solve problems effectively		0.79			
JS 23	The salary is commensurate with ability and dedication			1.02*		
JS 24	Occupational and toxic allowances are worthy of dedication			1.01*		
JS 25	Bonus and additional income are worthy of dedication			0.80		
JS 26	Fair distribution of bonus and other income sources			0.61		
JS 22	Fair distribution of the welfare funds			0.57		
JS 16	Friendly and united working environment				0.88	
JS 17	Colleagues share experiences and are willing to help each other at work				0.87	
JS 18	Colleagues care and help each other in daily life				0.84	
JS 15	Colleagues are willing to cooperate in common tasks completion				0.80	
JS 28	Adequate holidays and vacations/relaxation trips					0.95
JS 29	Adequate sports/arts movements and programs					0.91
JS 30	Active hospital union					0.70

Notes: Factors loading of less than 0.50 are not displayed.

*if the factors are correlated (oblique), the factor loadings are regression coefficients and not correlations and as such, they can be larger than one in magnitude.

**Table 2:** Descriptive analysis, reliability analysis, and logistic regression predicting factors affecting hospital pharmacists’ job satisfaction.

Factor	Factor name	Overall items	Mean (SD)	Median (IQR)	Satisfied*, n, (%)	Cronbach’s Alpha	Univariate analysis (p value)	Multivariate analysis			
								β (SE)	Wald	p value	OR (95%CI)
1	Working conditions	9	3.82 (0.61)	3.89 (0.67)	77.9	0.92	**<0.001**	2.10 (0.63)	11.20	**0.001**	8.16 (2.39–27.91)
2	Leadership styles	5	3.97 (0.60)	4.00 (0.80)	80.4	0.93	**<0.001**	1.91 (0.74)	6.74	**0.009**	6.74 (1.60–28.44)
3	Income	5	3.50 (0.77)	3.60 (1.00)	53.2	0.93	**<0.001**	0.79 (0.59)	1.77	0.183	2.20 (0.69–7.05)
4	Relationships with colleagues	4	3.92 (0.64)	4.00 (0.50)	81.3	0.92	**<0.001**	0.62 (0.62)	1.01	0.316	1.86 (0.55–6.22)
5	Benefits	3	3.61 (0.80)	4.00 (1.00)	57.9	0.89	**<0.001**	1.05 (0.54)	3.88	**0.049**	2.87 (1.00–8.18)
**Overall job satisfaction**		7	3.89 (0.60)	4.00 (0.57)	80.4	0.94		-	-	-	-

*Satisfied = average score of >3.4

β = regression coefficient; SE = standard error; OR = odds ratio; CI = confidence interval

The overall job satisfaction score was calculated by taking the average score across all the items on the satisfaction scale. Participants with a score greater than 3.4 were considered to have adequate job satisfaction, whereas those with less than or equal to 3.4 were deemed dissatisfied with their work [28]. Relationships between independent variables (sociodemographic and job-related characteristics) and dependent variable (job satisfaction) were evaluated using univariate and multivariate regression analyses. Variables exhibiting p < 0.05 in the univariate analysis were then included in the multivariate analysis. The odds ratio (OR) with 95% confidence intervals (CI) and significance level (p-value) was used to present numerical findings. The calibration of the multivariate regression model was assessed using the Hosmer-Lemeshow test. A non-significant p-value from the Hosmer-Lemeshow test suggests a satisfactory fit of the model. Additionally, logistic regression models were also performed to find out which of the five extracted factors were the best predictors of job satisfaction. The calibration of this logistic model was evaluated using the Hosmer-Lemeshow test and the area under the curve (AUC) [29–31]. Discrimination of the model was deemed acceptable if the AUC was ≥ 0.7. To address collinearity concerns in the multivariate analysis, it was essential to ensure that the selected variables had a Variance Inflation Factor (VIF) of 5 or lower (equivalent to a tolerance level of 0.2 or higher) [32].

### Inclusivity in global research

Additional information regarding the ethical, cultural, and scientific considerations specific to inclusivity in global research is included in the S1 Checklist.

### Ethical approval

The study was approved by the Medical Ethics Council of Can Tho University of Medicine and Pharmacy, Can Tho, Vietnam (reference: 22.019.HV/PCT-HDDD, 25th July 2022). Participants were informed that taking part in the study was voluntary. Survey responses were anonymized at source.

## Results

Of the 242 hospital pharmacists in the study population, 235 pharmacists completed the survey, representing a high response rate of 97.1%. The participants were from the Faculty of Pharmacy across all 17 province/district-affiliated healthcare facilities (7 public hospitals, 2 private hospitals, 1 city-affiliated medical centers, and 7 district-affiliated medical centers) in Vinh Long province (Fig 1).

**Fig 2:**
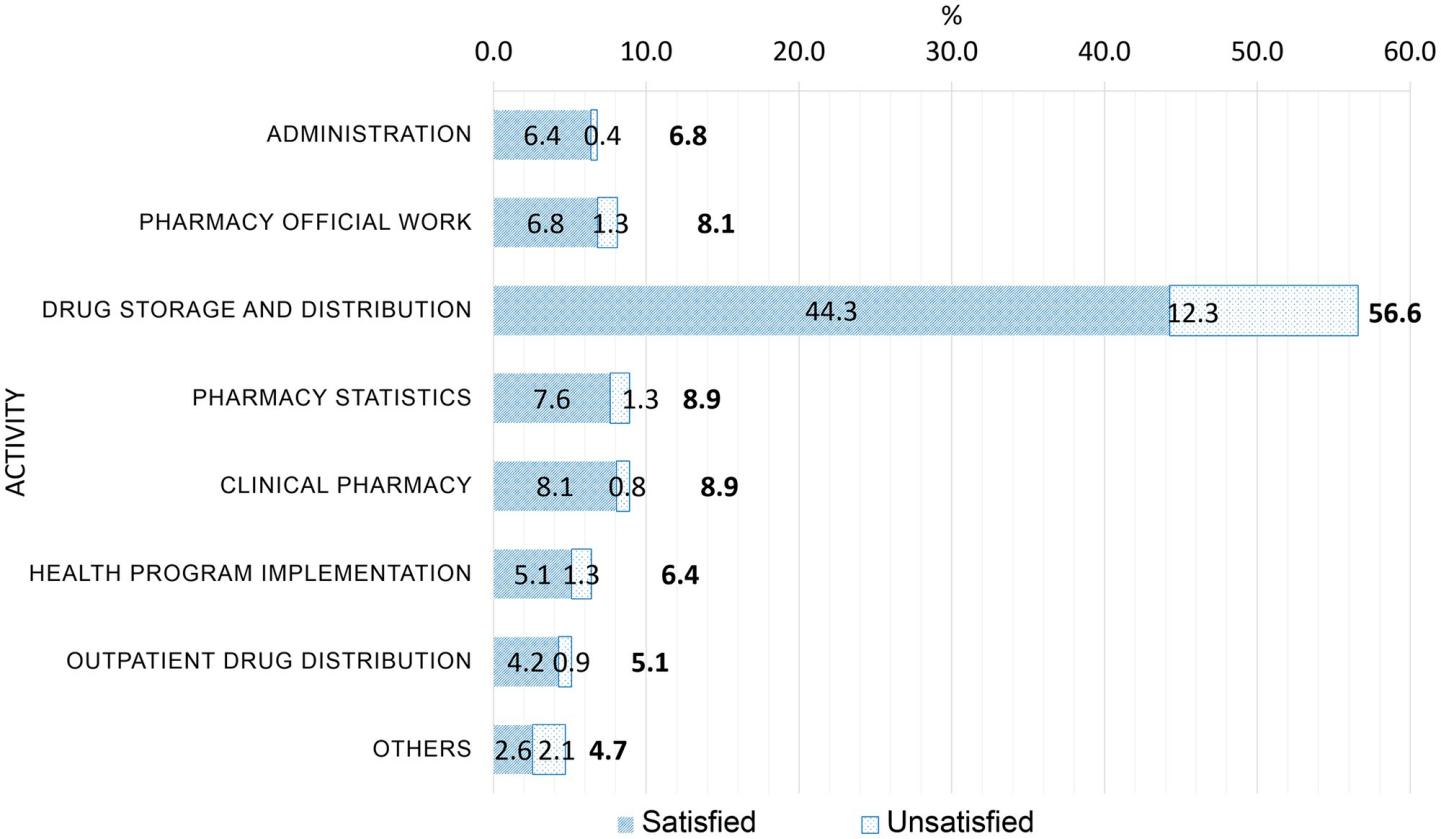
Satisfaction of the surveyed hospital pharmacists (in percentages) in Vinh Long, Vietnam, separated by job activity (n = 235). The relationship (Chi-squared test) between the drug storage/distribution activity and pharmacists’ satisfaction is p = 0.325. Other relationships were not determined due to the small percentages of responses.

Most respondents were female (74.5%), aged 30–40 years (60.0%), married (68.9%), had more than 3 family members (71.5%), and had achieved a bachelor’s degree or higher (59.6%) (Table 3). Most pharmacists worked in public healthcare facilities (86.8%), had worked in their field for more than 5 years (71.1%), did not have any additional duties besides their primary responsibilities (65.1%), were on duty < 4 times/month (89.8%), and received an average monthly income of ≥ 5 million VND (68.5%). Participants reported their workload including various activities including administration, pharmacy official work, drug storage and distribution, pharmacy statistics, clinical pharmacy, outpatient drug distribution. Drug storage and distribution was performed by more than half the participants (Fig 2).

**Table 3:** Sociodemographic and job-related characteristics of the participated hospital pharmacists, in terms of frequency (n) and percentage (%) (n = 235); and uni-/multivariate regression analyses on the relationships between these characteristics and the pharmacists’ job satisfaction. OR = odds ratio; CI = confidence interval.

Socio-demographic and job-related characteristic	n (%)	Job satisfaction		Univariate analysis			Multivariate analysis		
		No	Yes	OR	CI 95%	p	OR	CI 95%	p
**Gender**									
Man	60 (25.5)	29 (16.6)	146 (83.4)	1	-	**-**	1	-	-
Woman	175 (74.5)	17 (28.3)	43 (71.7)	1.990	1.000–3.962	**0.048**	1.764	0.845–3.682	0.131
**Age group**									
< 30 years	53 (22.6)	11 (20.8)	42 (79.2)	1	-	-			
30–40 years	141 (60.0)	27 (19.1)	114 (80.9)	1.106	0.504–2.425	0.802			
> 40 years	41 (17.4)	8 (19.5)	33 (80.5)	1.080	0.390–2.991	0.882			
**Accommodation type**									
Rental house	40 (17.0)	12 (30.0)	28 (70.0)	1	-	-			
Private house	195 (83.0)	34 (17.4)	161 (82.6)	2.029	0.939–4.387	0.072			
**Marital status**									
Single	73 (31.1)	16 (21.9)	57 (78.1)	1	-	-			
Married	162 (68.9)	30 (18.5)	132 (81.5)	1.235	0.625–2.442	0.544			
**Family members**									
≤ 3	67 (28.5)	14 (20.9)	53 (79.1)	1	-	-			
> 3	168 (71.5)	32 (19.0)	136 (81.0)	1.123	0.555–2.269	0.747			
**Average monthly income**									
< 5 million VND	74 (31.5)	19 (25.7)	55 (74.3)	1	-	-			
≥ 5 million VND	161 (68.5)	27 (16.8)	134 (83.2)	1.714	0.881–3.336	0.112			
**Working time**									
< 3 years	23 (9.8)	10 (43.5)	13 (56.5)	1	-	**-**	1	-	**-**
3–5 years	45 (19.1)	7 (15.6)	38 (84.4)	4.176	1.318–13.228	**0.015**	3.752	1.036–13.595	**0.044**
> 5 years	167 (71.1)	29 (17.4)	138 (82.6)	3.660	1.464–9.153	**0.006**	6.361	2.264–17.875	**< 0.001**
**Highest academic degree**									
College/Intermediate	95 (40.4)	15 (15.8)	80 (84.2)	1	-	-			
University/Bachelor	117 (49.8)	26 (22.2)	91 (77.8)	0.656	0.325–1.325	0.240			
Master/Specialist level I*	23 (9.8)	5 (21.7)	18 (78.3)	0.675	0.217–2.098	0.497			
**Presence of additional duties besides primary responsibilities**									
No	153 (65.1)	24 (15.7)	129 (84.3)	1.971	1.024–3.793	**0.042**	2.046	1.005–4.163	**0.048**
Yes	82 (34.9)	22 (26.8)	60 (73.2)	1	-	**-**	1	-	**-**
**Monthly on-duty times**									
< 4	211 (89.8)	40 (19.0)	171 (81.0)	1.425	0.532–3.820	0.481			
≥ 4	24 (10.2)	6 (25.0)	18 (75.0)	1	-	-			
**Overtime work**									
No	208 (88.5)	39 (18.8)	169 (81.3)	1.517	0.599–3.838	0.379			
Yes	27 (11.5)	7 (25.9)	20 (74.1)	1	-	-			
**Hospital type**									
Public	204 (86.8)	45 (22.1)	159 (77.9)	1			1	-	-
Private	31 (13.2)	1 (3.2)	30 (96.8)	8.491	1.127–63.983	**0.038**	12.021	1.470–98.316	**0.020**

*Pharmacy staff who graduated 2-year postgraduate practical/clinical training program in pharmacy education.

The results of the univariate analysis describing the relationship between sociodemographic/job-related characteristics and job satisfaction showed that gender, working years, presence of additional duties besides primary responsibilities, and hospital type were the four main factors contributing to the job satisfaction (Table 3). The multivariate regression analysis demonstrated that working years, presence of additional duties besides primary responsibilities, and hospital type were statistically associated with job satisfaction. Pharmacists who had worked in the field for 3–5 years (OR = 3.752, 95% CI = 1.036–13.595), more than 5 years (OR = 6.361, 95% CI = 2.264–17.875), did not have any other duties (OR = 2.046, 95% CI = 1.005–4.163), or worked in private hospital (OR = 12.021, 95% CI = 1.470–98.316) had significantly higher job satisfaction than other groups. This model was a good fit as indicated by Hosmer and Lemeshow test (χ^2^ = 2.870, df = 7, p = 0.142) and area under the ROC curve was 0.729 (95% CI = 0.652–0.806, p < 0.001).

Analysis of the logistic regression model showed that three out of five factors statistically affected the pharmacist job satisfaction. These included working conditions, leadership styles, and benefits (Table 2). Of these, working conditions were the most strongly contributing factor (OR = 8.16, 95% CI = 2.39–27.91). This model was a good fit as indicated by Hosmer and Lemeshow test (χ^2^ = 10.915, df = 7, p = 0.142) and area under the ROC curve of 0.967 (95% CI = 0.947–0.986, p < 0.001). The VIF values and tolerance levels of the five factors ranged from 2.05 to 2.57 and from 0.39 to 0.49, respectively, indicating the absence of multicollinearity in the data.

## Discussion

Of the 235 pharmacists working at 17 province/district-affiliated healthcare facilities in Vinh Long province, the greatest proportion of them had university degrees (49.8%), and those with a master’s degree or specialist level I qualifications only accounted for 9.8% of respondents, in agreement with the findings of Nguyen Thi HY et al. [28]. This could be related to the report that in 2020, only about 830 pharmacists across Vietnam had attained master’s degrees, according to the statistics of the Vietnam Ministry of Health [33]. Furthermore, there were only 21 clinical pharmacists across 17 healthcare facilities (~1.23 clinical pharmacists per healthcare facility), a figure lower than that in Nguyen Thi HY’s study, which reported 187 clinical pharmacists in 79 participating hospitals (~2.37 clinical pharmacists per hospital) [5]. Consequently, it is crucial to investigate the job satisfaction of pharmacists so as to make improvements to enhance the productivity of this limited workforce, as job satisfaction has been positively assciated with performance, productivity, management relationships, and physical and mental health [34].

The overall job satisfaction of hospital pharmacists in our study was 80.4%, which was higher than that reported in Ho Chi Minh city, Vietnam (74.1%) [28], Malaysia (52%) [35], and Ethiopia (53%) [34], but lower than that reported in China (90%) [36] and Japan (95%) [37]. Our data re-confirms that job satisfaction varies widely across countries, and depends on various external and internal factors [21]. Additionally, the differences in the questionnaires and the cut-off points used to categorize job satisfaction may also contribute to the wide variation observed across countries. Specifically, in the present study, factor analysis identified 5 main factors affecting job satisfaction, including working conditions (9 items), leadership styles (5 items), income (5 items), relationships with colleagues (4 items), and benefits (3 items). Of these, working conditions contributed most strongly to job satisfaction (OR = 8.16, 95% CI = 2.39–27.91). Hospital pharmacists who experienced a congenial working condition were eight times more satisfied with their jobs than those who did not. Previous studies have demonstrated that a favorable working environment facilitates professional competence and job satisfaction in healthcare workers [35, 38, 39]. Furthermore, employees have used poor working conditions as an excuse for bad performance [35, 39, 40]. Therefore, managers should pay attention to both working conditions and performance when addressing the job satisfaction of hospital pharmacists.

The second factor that greatly contributed to job satisfaction was the leadership styles (OR = 6.74, 95% CI = 1.60–28.44). The impact of leadership on employees’ satisfaction has been discussed for various healthcare-related roles [41–44]. The present findings suggest that for hospital pharmacists, the impact of leadership may be even more significant. Hospital pharmacists are most satisfied when their leaders consider complaints and respond in a timely manner. They have higher job satisfaction when they feel that their leader is responsible, fair, understands the employee’s job function [44], and supports and recognizes the employee’s contribution [45]. Therefore, practicing active leadership strengthens the leaders’ ability to be effective and proficient, consequently increasing the job satisfaction of hospital pharmacists.

The third contributing factor was found to be benefits (OR = 2.87, 95% CI = 1.00–8.18), including the items of “Adequate holidays and vacations/relaxation trips”, “Adequate sports/arts movements and programs”, and “Active hospital union”. Insufficient benefits causes a work-life imbalance, thereby reducing job satisfaction [39], since employees are unable to fulfil their job requirements if their own needs are not satisfied [46]. This factor is also important for addressing differences in job satisfaction between pharmacists working in public and private hospitals, because pharmacists working in private hospitals tend to report that their working conditions are beneficial, while those in public hospitals highly appreciate any benefits they receive [20].

Although income has been demonstrated to significantly affect healthcare workers’ satisfaction, as low-wage hospital pharmacists who were less satisfied with their jobs being more likely to leave their jobs for higher-income ones, compared to others [19], our results demonstrated no significant effect of income on job satisfaction. Our findings also contradict those of a study conducted by Nguyen Thi HY in Ho Chi Minh city, Vietnam, which reported income to be a significant factor affecting job satisfaction [5]. This could be due to socioeconomic differences between participants in the two studies. Ho Chi Minh city is the most populous city in Vietnam, with a high living cost, thus elevating the importance of income, especially for pharmacists working in the public sector, who receive a fixed-rate monthly salary (salaries issued by the Vietnam government are fixed for everyone working at a given levels of expertise, regardless of working location). Conversely, the cost of living in Vinh Long is not as high, meaning that pharmacists earning the standard rate can easily afford their monthly expenses. Consequently, to equalize the living conditions of hospital pharmacists across Vietnam, and improve job satisfaction, policymakers should consider adjusting the existing fixed-rate salary scale based on the cost of living in different areas of Vietnam.

Relationships between sociodemographic characteristics and job satisfaction are shown in Table 3. Women pharmacists were more satisfied with their work than men (p < 0.05), in agreement with previous studies [35, 47, 48]. This is consistent with analogous research conducted in other healthcare-related disciplines, which has demonstrated that men and women display different attitudes towards jobs and practices in the development of society, consequently affecting their perceptions on job satisfaction [49]. Similarly, pharmacists who had been working in their field for longer time were more satisfied with their work than those who had shorter careers, in accordance with previous findings [35, 50, 51]. This may be due to senior pharmacists being more competent and efficient, making their daily workload easier, giving them greater confidence and satisfaction in their profession [35]. Conversely, newcomers in this field often have less experience, face challenges in adapting and developing, deal with competition, and have uncertainty about their career goals, leading to lower job satisfaction. However, young pharmacists seem to be actively seeking more professionally satisfying roles [52]. Additionally, hospital pharmacists who did not have additional duties besides their primary responsibilities were more satisfied than those who did (OR = 2.046, 95% CI = 1.005–4.163, p = 0.048). Evidently, as workload is an important factor dictating job satisfaction [47, 53], an increased workload leads to increased stress levels, and a reduction in job satisfaction [7, 34, 54, 55]. Therefore, hospital managers should allocate activities in a way that does not add to the workload of staff. Finally, pharmacists working in private hospitals were more satisfied with their jobs compared to those working in public hospitals. This was in agreement with previous studies in Zambia [56] and South Africa [20]. To equalize working conditions, benefits, and job satisfaction between the private and public sectors, it is imperative that policymakers pay more attention and take critical action to improve the experiences of healthcare workers in the public sector.

### Study limitations

Although successfully addressing the research question, this study has some limitations. Firstly, all data were collected using questionnaires completed unsupervised by participants, and thus, may not be free from bias. To try to prevent this, participant responses were anonymised at source and participants were assured of the anonymity of their responses. Secondly, this study employed a non-random sampling procedure, which may introduce bias and limit the generalizability of the study’s results. Thirdly, these findings cover a representative area of Southern Vietnam and report the experiences of healthcare professionals in that area only and will provide useful insight for provinces/cities with similar socioeconomic conditions and models to Vinh Long. However, these findings may not be highly generalizable to the whole Vietnam and larger/higher-income countries outside of Southeast Asia. Fourthly, the factor loadings of JS 23 and JS 24 exceeding 1.0, as explained by Jöreskog, “if the factors are correlated (oblique), the factor loadings are regression coefficients and not correlations and as such they can be larger than one in magnitude” [57]. This may not undermine the validity of our study’s findings [58]. These variables were retained because they contributed to the interpretation of the income factor, which was consistent with the findings of Nguyen Thi HY et al. [28]. Further research should be conducted to thoroughly assess their impact on the observed outcomes. Finally, wider confidence intervals were observed in private facilities for both univariate and multivariate analyses, likely due to the smaller sample size of hospital pharmacists in private facilities within this study, resulting in the potential presence of unmeasured or uncontrolled factors unique to private facilities. These findings emphasize the need for careful interpretation and generalization of the results pertaining to this variable. Further research is warranted to gain a deeper understanding of the underlying factors contributing to the observed disparities between private and public facilities.

## Conclusions

The study is the first to provide insights on the job satisfaction of hospital pharmacists working across all province/district-affiliated healthcare facilities in Vinh Long province, Vietnam. In conclusion, the majority of hospital pharmacists were satisfied with their current jobs. Factors that were significantly associated with better job satisfaction included working conditions, leadership styles, and benefits, in which improving working conditions was the most influental factor in the level of job satisfaction. Consequently, it is recommended that the hospital pharmacy managers and policymakers should address and improve workload models in hospital pharmacy departments, improving workload transparency, working environments, employees’ ability to fulfil their potential and achieve promotion, and the associations between workload, income, and benefits.

## Supporting information

S1 Checklist (DOCX)
