# Correction: Job Satisfaction of Hospital Pharmacists in a representative province in Mekong Delta, Vietnam

**DOI:** 10.1371/journal.pone.0297955

**Published:** 2024-01-25

**Authors:** Van De Tran, Thi My Loan Vo, Khanh Nguyen Di, Quang Loc Duyen Vo, Rebecca Susan Dewey, Trung Tin Pham, Ba Kien Tran, Duy Toan Pham

The affiliation for the eighth author is incorrect. Duy Toan Pham is not affiliated with #4 but with #5: Department of Chemistry, College of Natural Sciences, Can Tho University, Can Tho, Vietnam.
